# Production, performance and emission of biodiesel from a mixture of castor oil and neem oil

**DOI:** 10.1039/d5ra04004c

**Published:** 2025-09-24

**Authors:** M. Arslan, Hamid Ayyub, M. Jamshaid, A. Arslan, M. A. Kalam, Farah Ahmad

**Affiliations:** a Department of Mechanical Engineering, Faculty of Engineering and Technology, Bahauddin Zakariya University 60800 Multan Pakistan muhammad.jamshaid@bzu.edu.pk; b Department of Mechanical Engineering, COMSATS University Islamabad 46000 Wah Campus Pakistan; c School of Civil and Environmental Engineering, FEIT, University of Technology Sydney Ultimo NSW 2007 Australia; d Department of Chemical Engineering and Sustainability, Faculty of Engineering, IIUM 53100 Kuala Lumpur Malaysia

## Abstract

The elimination of reserves of petroleum and their consequential environmental impact prompts the development of alternative fuels. This study aimed to blend castor and neem oils (at an 80 : 20 ratio) to address the drawbacks present in castor oil biodiesel, such as elevated kinematic viscosity and density. We propose that this new blending with a highly effective heterogeneous calcium oxide catalyst is the novelty of this work. This study employed a response surface approach to optimize biodiesel production. Biodiesel blends (B10, B20, and B30) were examined *via* standards EN 14214 and ASTM D6751. The performance of the biodiesel blends was scrutinized under experimental conditions, operating at a steady 2000 rpm with engine loads in the 25–100% range. Biodiesel production was optimized at an 8.75 : 1 methanol-to-oil ratio, 3.01 wt% calcium oxide, 56.6 °C, and 800 rpm, achieving a 95% methyl ester yield. The engine performance results indicated that brake thermal efficiency was lower than that of petroleum diesel. Conversely, brake-specific fuel consumption exhibited higher values than those observed with petroleum diesel. In terms of emissions, carbon monoxide and smoke opacity were less common than when using petroleum diesel, as the average smoke opacity for diesel was 10.46%, 18.43%, and 26.93% greater than that of the B10, B20, and B30 blends, respectively. However, the carbon dioxide and nitrogen oxide emissions were greater than those of petroleum diesel. Thus, a biodiesel blend from castor and neem oils can be a viable substitute fuel for internal combustion engines.

## Abstract 2

The elimination of reserves of petroleum and their consequential environmental impact prompts the development of alternative fuels.

## Graphical abstract

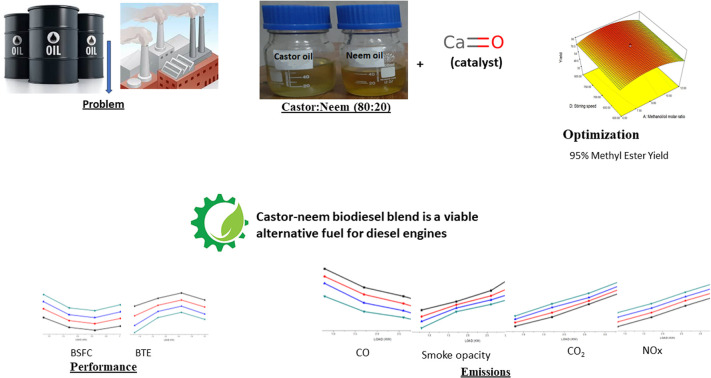

## 1. Introduction

Fossil fuels are crucial for industrial growth, but their excessive use depletes nonrenewable resources and contributes to global warming. Growing environmental concerns have driven extensive research on alternative fuels.^1^ Researchers are developing more efficient ways to produce energy from green and renewable sources,^2^ including solar, wind, hydro, and tidal sources. Biofuels such as biodiesel, biogas, and bioethanol provide an optimistic mode to fulfill future global energy needs.^3^ The triglycerides found in vegetable oils react with alcohols and other substances to create fatty acid esters known as alkyl esters, which are the building blocks of biodiesel.

Biodiesel, a biodegradable alternative fuel, emits fewer greenhouse gases than petroleum diesel does.^4^ First-generation feedstocks include edible oils such as canola, soybean, palm, and rapeseed, which are favored for their low FFA content and high yield with alkali catalysts.^5^ Feedstock costs account for 70–80% of biodiesel production, making nonedible oils a viable, cost-effective alternative.^6^ Mengistu *et al.*^7^ investigated the process of transesterification of castor oil with heterogeneous catalysts made from animal waste and a combination of teeth and bone. They attained a biodiesel yield of 92.6%. Nurdin *et al.*^8^ investigated the conversion of castor oil into biodiesel by using a heterogeneous catalyst made from calcined mussel shells, and a 91.17% yield was obtained. In another study, Noreen *et al.*^9^ produced biodiesel from neem oil *via* the use of Ni-, Fe-, and Cu-doped ZnO as heterogeneous catalysts. 80%, 95%, and 85% biodiesel were produced. The work of Ulakpa *et al.*^10^ contributed to biodiesel production from neem oil with the utilization of waste bone as a heterogeneous catalyst, achieving a 94% biodiesel yield.

Researchers have combined various feedstocks, including edible and nonedible oils, to address feedstock shortages and fuel quality issues. M. A. Mujtaba *et al.*^11^ achieved a 96.61% biodiesel yield by transesterification equal parts of palm and sesame oils with methanol. T. F. Adepoju *et al.*^12^ achieved a 98.03% biodiesel yield *via* a 60 : 40 blend of pig and neem seed oils. Another investigation by S. Niju *et al.*^13^ achieved a 96.5% conversion rate by blending *Calophyllum inophyllum* oil with waste cooking oil (WCO).

Catalysts, with enzymes, homogeneous, and heterogeneous types, are essential in biodiesel production.^14^ Heterogeneous catalysts offer high activity under mild conditions and are easier to separate and reuse.^15^ S. R. Dasari *et al.*^16^ undertook experimental investigations to examine diesel mixtures with castor oil biodiesel, including COME15, COME10, and COME5, to conduct numerous engine performance tests. Among these blends, B10 (a blend containing 10% COME) exhibited the highest brake thermal efficiency when the engine was operating at the maximum load. Significant reductions in CO (26–30%), HC (17.5–50%), and NOx (14–20%) exhaust gas emissions were observed when the quantity of COME increased in petroleum diesel. J. N. Nair *et al.*^17^ used neem oil to make biodiesel, and engine tests were conducted using various blends, namely, B10, B20, and B30, in combination with diesel fuel. The results indicated that B10 had the lowest emissions and highest performance compared with those of pure diesel and the other mixture ratios. Moreover, B10 had the highest BTE, and the emissions of CO and HC decreased by 8.55% and 23%, respectively. B. A. Oni *et al.*^18^ conducted an experimental study and scrutinized various characteristics (emissions and performance) of biodiesel blends acquired *via* neem and camelina sativa oil. The diesel engine was tested using numerous fuel mixtures, including B10, B5, and pure diesel. Engine performance tests revealed that CB10 biodiesel displayed better BP and relatively higher BSFC than did pure diesel fuels. Noticeable reductions in CO emissions were observed for NB5 (4.84%), CB5 (8.79%), NB10 (10.77%), and CB10 (12.09%). However, higher NOx emissions were observed for NB5 (18.7%), CB5 (3.14%), NB10 (19.33%) and CB10 (19.78%) than for pure diesel fuel. Research by M. Jamshaid *et al.*^19^ revealed a low BTE for various amalgamated biodiesel mixtures, including C15P05, C10P10, and C05P15, and lower emission levels of HC, CO, and smoke opacity. However, slightly increased NOx levels were found compared with those in pure diesel. S. Arunprasad *et al.*^20^ used a biodiesel mixture originating from nonedible oils. Their findings revealed notable outcomes, including a 28.1% brake thermal efficiency (BTE) enhancement. However, there was an increase of 31.47% in NOx emissions and a substantial 54.1% increase in CO2 emissions. Gowtham *et al.*^21^ studied blended biodiesel composed of a mixture of Pongamia and coconut oils to determine its emission and performance characteristics. The findings indicated that when a B40 blend of this biodiesel was used, there was a reduction of 2.4% in BSFC and a 6.2% decrease in BTE compared with conventional diesel fuel.

Ahmed Mohammed Elbanna *et al.*^22^ injected a diesel/ethanol mixture (75% diesel, 25% ethanol by volume) into a combustion chamber. Tests on the engine showed that the BSFC was lowered by 8–15%, the UHC emission was reduced by 52%, the CO emissions were decreased by 41%, and ultralow nitrogen oxide (NOx) (below 1 g kW^−1^ h^−1^) was detected in the case of direct dual-fuel stratification (DDFS). Research by Mostafa M. El-Sheekh *et al.*^23^ revealed that the operating parameters of a single-cylinder DI engine running on a blend of 50% biodiesel/50% diesel with 10% and 20% bioethanol were optimized *via* the central composite design approach (CCD) to achieve the highest possible break thermal efficiency (BTE%) and lowest NOx emissions. The research by Hagar Alm El-Din *et al.*^24^ aimed to validate the potential of using dimethyl ether (DME) as an additive in a blend of pure natural gas or a natural gas/hydrogen blend to increase the performance, efficiency, and emission of a homogeneous charge compression ignition (HCCI) engine. The main aim was to find the optimal operating conditions of a CNG HCCI engine with minimum numbers of laboratory engine tests.

While biodiesel production from non-edible oils and heterogeneous catalysts has been widely studied, our research focuses specifically on blending castor oil (Ricinus communis) and neem oil (Azadirachta indica) at an 80 : 20 ratio. Castor oil, despite its advantages such as high hydroxyl value and solubility in alcohols, produces biodiesel with elevated viscosity and density.^25^ Neem oil, rich in triglycerides and triterpenoid compounds with saturated and unsaturated fatty acids,^26^ was selected to offset these drawbacks. The novelty of this study lies in demonstrating that blending castor and neem oils, together with an efficient heterogeneous calcium oxide catalyst, can overcome the limitations of castor-based biodiesel. The biodiesel yield was evaluated by examining the impact of various process input parameters. These parameters included the catalyst dosage, alcohol-to-oil molar ratio, reaction temperature, and stirring speed. Fuel properties such as density, kinematic viscosity, cetane number, acidic value, and calorific value of the castor oil and neem oil mixture were investigated and compared with the EN 14214 and ASTM D 6571 standards. This investigation involved examining engine performance and emission aspects, such as BTE, EGT, BSFC, CO, CO_2_, NOx_,_ and smoke opacity, when a blend of castor and neem oils is used. It contributes to reducing greenhouse gases and offers an affordable replacement for fossil fuels.

## 2. Materials and methods

### 2.1. Materials

**Fig. 1:**
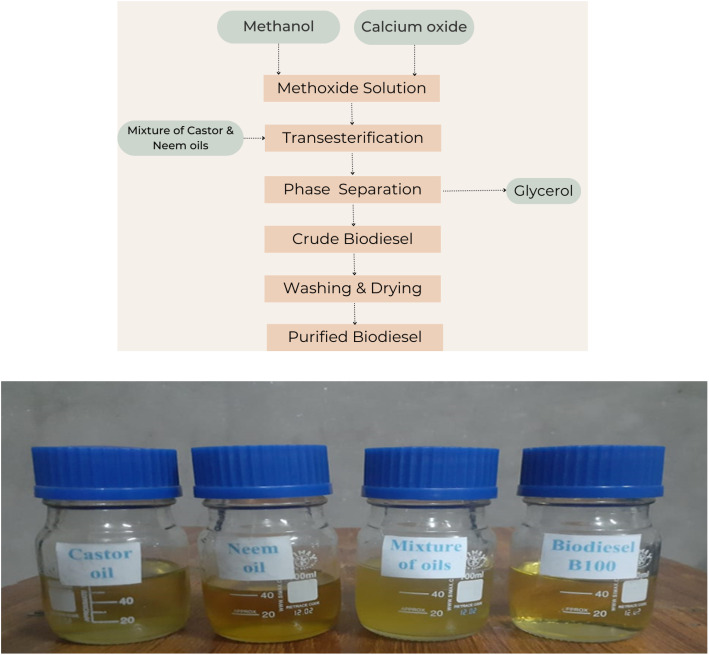
(a) Biodiesel production steps. (b) Samples of nonedible oils and biodiesel.

Castor and neem oils were sourced from Multan, Pakistan. Methanol (99% pure), calcium oxide (catalyst), and anhydrous sodium sulfate for biodiesel production were obtained from local suppliers. The transesterification process was carried out *via* the steps mentioned in Fig. 1(a). The acidity of the castor and neem oil mixture was 4.36 mg KOH per g of oil.

### 2.2. Preparation of the oil mixture

In this work, a blend of two distinct oils, namely, castor oil and neem oil, was taken at a ratio of 80 : 20. The blending process was carried out *via* a magnetic stirrer hot plate, which was run for 30 minutes at 650 rpm to ensure that the oils were thoroughly mixed and that a uniform mixture was achieved. Once a homogeneous blend is obtained, it serves as the input needed to make biodiesel.^27^

### 2.3. Transesterification process

The homogeneous oil mixture was preheated for transesterification *via* a specialized apparatus, as shown in Fig. 1. Each experiment used 500 g of oil, which was heated before a methoxide solution, which was formed by mixing calcium oxide with methanol, was added. The reaction was conducted at a controlled speed for 120 minutes. After cooling and resting for 24 hours, the mixture was separated into two layers: glycerol (bottom) and biodiesel (top). The glycerol was removed *via* a separating funnel.

To purify the crude biodiesel, a 20% distilled water wash was performed 3–4 times to eliminate glycerol, unreacted methanol, and excess catalyst. The remaining water was removed with anhydrous sodium sulfate, followed by filtration, yielding the final biodiesel product. The biodiesel yield was calculated as the ratio of the biodiesel weight (g) to the oil weight (g) in the sample multiplied by 100%.

### 2.4. Gas chromatography analysis

Gas chromatography is a widely used method for analyzing mixture compositions.^28^ A gas chromatography (GC) analyzer (Shimadzu 2014) was employed to determine the FAME composition of the oils and produced biodiesel. Both the injection and detector temperatures were set at 300 °C to ensure complete vaporization of the sample and prevent condensation during detection. Nitrogen, an inert gas, served as the carrier, supporting stable operation with a flame ionization detector (FID). A 1 μL sample was injected in separate mode, likely split or splitless, to control sample introduction on the basis of analyte concentration. The carrier gas flowed at a fixed rate, maintaining consistent retention times. A capillary column (0.53 mm × 30 m, 0.5 μm film) provided efficient separation. The FID detected organic compounds by measuring ionized carbon fragments generated in a hydrogen-air flame, offering high sensitivity for both qualitative and quantitative analysis.

### 2.5. Design of the experiment

The central composite design (CCD) was used to optimize the transesterification of castor and neem oils by precisely adjusting variables while minimizing trials.^29^ In this study, the CCD utilized one response variable and four input factors: the methanol/oil molar ratio, temperature, stirring speed and catalyst concentration, as shown in Table 1. The biodiesel yield from the transesterification process served as the response variable. Table 2 summarizes the input variables, their units, and value ranges.

**Table 1:** Design of experiments for four input factors and one response variable

Run	Point type	*A*: Methanol/oil molar ratio (mol mol^−1^)	*B*: Temperature (°C)	*C*: Heterogeneous catalyst (wt%)	*D*: Agitation speed (rpm)	Biodiesel yield (%)
1	Axial	9	45	2.75	650	61
2	Axial	9	55	0.75	650	54
3	Center	9	55	2.75	650	91
4	Axial	9	55	4.75	650	74
5	Factorial	6	50	1.75	800	64
6	Factorial	12	50	1.75	500	57
7	Factorial	12	60	1.75	500	62
8	Center	9	55	2.75	650	93.5
9	Factorial	6	50	1.75	500	62
10	Factorial	12	50	3.75	800	60
11	Factorial	12	60	1.75	800	67
12	Factorial	12	50	1.75	800	60
13	Axial	3	55	2.75	650	45
14	Factorial	6	60	3.75	800	77
15	Factorial	6	50	3.75	500	65
16	Factorial	6	50	3.75	800	68
17	Axial	9	65	2.75	650	73
18	Factorial	12	50	3.75	500	58
19	Factorial	12	60	3.75	800	72
20	Center	9	55	2.75	650	93
21	Axial	15	55	2.75	650	37
22	Center	9	55	2.75	650	90
23	Factorial	6	60	1.75	500	66
24	Factorial	6	60	1.75	800	68
25	Center	9	55	2.75	650	92.5
26	Factorial	12	60	3.75	500	70
27	Center	9	55	2.75	650	90.5
28	Axial	9	55	2.75	350	85
29	Axial	9	55	2.75	950	95
30	Factorial	6	60	3.75	500	75

**Table 2:** Experimental design for biodiesel synthesis

Independent factors	Units	Levels^a^				
		−1	+1	Center	−Alpha	+Alpha
*A*-Methanol/oil molar ratio	(mol mol^−1^)	6	12	9	3	15
*B*-Temperature (°C)	(°C)	50	60	55	45	65
*C*-Heterogeneous catalyst conc.	(wt%)	1.75	3.75	2.75	0.75	4.75
*D*-Stirring speed	(rpm)	500	800	650	350	950

a Each numeric factor is varied over 5 levels: plus and minus alpha (axial points), plus and minus 1 (factorial point), and the center point.

### 2.6. Statistical analysis

The experiments were designed with the program used for the experimental data, State-Ease 360. Eqn (1) was used to calculate yield considering the four input factors and their interactions.1
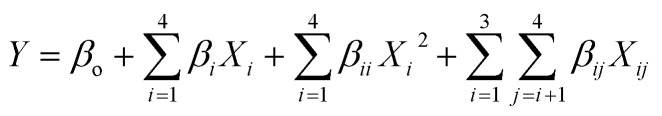
*Y* stands for the expected production of biodiesel, *X*_*i*_ is the input factor for *i*th, and different coefficients such as *β*_o_ (intercept), *β*_*i*_ (the first-order model's coefficients), *β*_*ii*_ (coefficients for a quadratic model of each input factor), and *β*_*ij*_ (coefficients between different input factors) are utilized within the equation. The independent variables' values and their interactions' statistical significance were determined *via* analysis of variance (ANOVA). ANOVA was used to consider experimental variation. To evaluate the independent variables' statistical significance, their interactions, and the fit quality of the fitted model, an ANOVA was employed.

### 2.7. Determining the properties of biodiesel

Biodiesel properties, including calorific value, density, flashpoint, cloud point, pour point, kinematic viscosity, acid value, and water content, were evaluated *via* ASTM standard procedures, as detailed in Table 3.

**Table 3:** Standard methods and testing apparatus for determining fuel properties

Sr./no.	Properties	ASTM methods	Testing equipment
1	Kinematic viscosity at 40 °C (mm^2^ s^−1^)	D-445	Cannon viscometer
2	Calorific value (MJ kg^−1^)	OEM	Bomb calorimeter
3	Acidic no. (mg KOH per g)	D-974	Titration
4	Flash point (°C)	D-92	Open cup cleavland
5	Pour point (°C)	D-97	Pour point apparatus
6	Fire point (°C)	D-92	Open cup cleavland
7	Fire point (°C)	D-92	Open cup cleavland
8	Fire point (°C)	D-976	Portable cetane/octane meter

### 2.8. Tested fuel preparation

Four fuel variations were read for experimentation in the 4-stroke diesel engine. Among these, one was singled out petroleum diesel (D100). The remaining trio consisted of distinct combinations derived from petroleum diesel and biodiesel. These mixtures were designated B10 (90% petroleum diesel and 10% biodiesel by volume), B20 (80% petroleum diesel and 20% biodiesel by volume), and B30 (70% petroleum diesel and 30% biodiesel by volume). These three mixtures were meticulously prepared on the basis of their volumetric proportions. The ensuing phase encompassed performance and emission assessments of all four fuel variations *via* the single-cylinder 4-stroke diesel engine setup.

### 2.9. Engine test

**Fig. 2:**
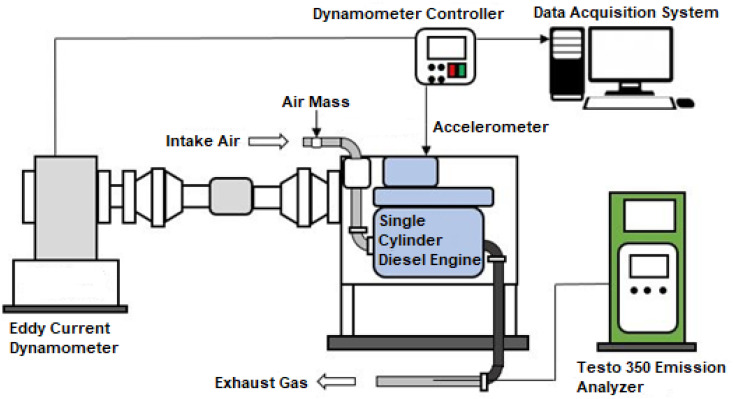
Schematic diagram of a single-cylinder diesel engine.

This section examines how biodiesel from nonedible oils affects a single-cylinder, four-stroke diesel engine's performance and emissions. The engine testing occurred in the internal combustion (IC) Engine laboratory at Bahauddin Zakariya University in Multan. A TD200 eddy current dynamometer was used to measure the torque, which was coupled to a diesel engine. In Fig. 2, the engine arrangement is shown. A K-type thermocouple was used to monitor the temperatures of the inlet air and exhaust gas. Table 4 provides the engine specifications. Diesel engine performance and the impacts of adding biodiesel in various ratios with diesel were studied. This included factors such as BSFC and BTE. Additionally, this study assessed how this blending influences the emission of different pollutants, including smoke opacity, NOx, CO, and CO_2_. Engine testing involved the use of different fuel mixtures, including D100, B10, B20 and B30. The emission analysis and measurement of CO, CO_2_, and NOx emissions were conducted with a Testo 350 emission analyzer.

**Table 4:** Engine features in the laboratory layout

Sr./no.	Specification	Values
1	Number of cylinders	1
2	Connecting rod length (mm)	104
3	Maximum torque (Nm)	11
4	Maximum power (kW)	3.5
5	Comp. Proportion	22 : 1
6	Maximum revolution per minute	3019
7	Bore & stroke (mm)	69 & 62

To control fuel flow as needed, a two-way valve was integrated into the fuel line system. Blended biodiesel fuel was stored separately from petroleum diesel fuel. The engine was initially operated with petroleum diesel until it stabilized. Subsequently, blended fuels were introduced into the engine. A 15-minute running period with blended fuel was employed to purge any remaining petroleum diesel from the engine. Before each test, the parameter readings were validated by the data acquisition system. To ensure repeatability, every test was run three times with the same blended fuel, under identical conditions, within a short time frame, and with the same equipment and operator. Once every test was finished, the engine was run using petroleum fuel, ensuring the removal of any remnants of the previously tested blended fuel. This consistent approach was applied to all the different blends. A frequency meter was used to track the engine speed during the test, which was maintained at 2000 rpm. This study considered 25%, 50%, 75%, and 100% engine loadings. Eqn (2)–(4) were used to compute BP, BSFC, and BTE. Previous studies established a notable correlation between vehicle emissions and specific fuel consumption, which was used to measure emissions in g kW^−1^ h^−1^,^30^ as shown in eqn (5).

2 BP = (2π*N*/60) × *T*

3 BSFC = *ṁ*/BP

4 BTE = (3600/BFSC × CV) × 100%

The engine speed is expressed as *N* (rpm), the torque obtained is expressed as *T* (Nm), the fuel mass flow rate is expressed as *ṁ* (g h^−1^), and the fuel calorific value is expressed as CV (MJ kg^−1^).

5 *E*_Pi_ = *E*V_*i*,d_ × [*M*_*i*_/*M*_Exh,d_ × *m*_Exh,d_/*P*_eff_] = EV_*i*,w_ × [*M*_*i*_/*M*_Exh,w_ × *m*_Exh,w_/*P*_eff_]

where *E*_Pi_ (g kW^−1^ h^−1^) is the pollutant mass, *M*_*i*_ (g mol^−1^) is the molecular mass of the components, and *M*_Exh,d_ (g mol^−1^) and *M*_Exh,w_ (g mol^−1^) are the molecular masses of exhaust gases on dry and wet bases, respectively. EV_*i*,d_ (ppm) and EV_*i*,w_ (ppm) denote exhaust emission values on dry and wet bases, respectively, whereas *m*_Exh,d_ (kg h^−1^) and *m*_Exh,w_ (kg h^−1^) indicate exhaust mass flow on dry and wet bases, respectively. Finally, *P*_eff_ (kW) represents the power output.

## 3. Results and discussion

### 3.1. Regression model equation

The experimental biodiesel yield, as detailed in Table 2, ranged from 37% to 95%. These coefficients were then applied to eqn (1). Eqn (6) displays the needed quadratic models as coded units.

6 *Y* = +91.75 − 2.29167 × *A* + 3.625 × *B* + 3.29167 × *C* + 1.70833 × *D* + 0.5625 × *A* × *B* − 0.6875 × *A* × *C* + 0.1875 × *A* × *D* + 1.4375 × *B* × *C* + 0.0625 × *B* × *D* − 0.1875 × *C* × *D* − 12.6562 × *A*^2^ − 6.15625*B*^2^ − 6.90625*C*^2^ − 0.40625*D*^2^

**Fig. 3:**
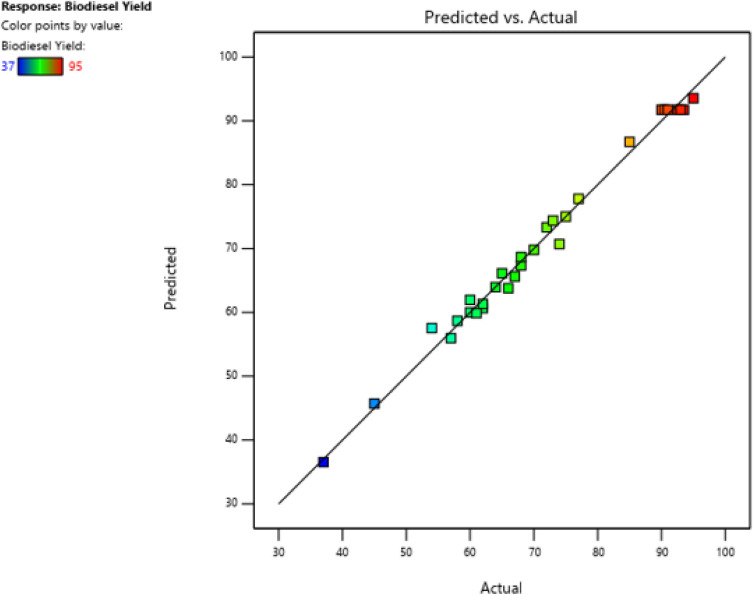
Comparison of the predicted and actual yields (%).

The independent input factors selected for the transesterification process were responsible for 98.06% of the variation in the results, according to the model's correlation value (*R*^2^) of 0.9906. Ideally, a high degree of agreement between the experimental and predicted results is indicated by an *R*^2^ value of 1. The correlation between the predicted and experimental findings based on the constructed model is demonstrated in Fig. 3.

### 3.2. Statistical evaluation of the production process

Table 5 presents the results of the response surface quadratic model analyzed *via* analysis of variance (ANOVA).

**Table 5:** ANOVA for the quadratic model showing the mean square, *F* value, *p* value, and significance

Source	Mean square	*F* Value	*p* Value	
Model	464.60	113.09	<0.0001	Significant
*A*-Methanol/oil molar ratio	1260.04	30.68	<0.0001	
*B*-Temperature	315.38	76.76	<0.0001	
*C*-Catalyst concentration	260.04	63.30	<0.0001	
*D*-Stirring speed	70.4	17.05	0.0009	
Residual	4.11			
Lack of fit	5.13	2.47	0.1651	Not significant
Pure error	2.08			

The model's strength is supported by a high Model *F* value of 113.09, suggesting a less than 0.01% chance that this result is due to random variation. The significance of each coefficient is indicated by its *p* value, with model terms considered insignificant if their *p* value exceeds 0.100. A lack of fit *F* value of 2.47 indicates no significant deviation from the actual data. Table 6 reports the model's statistical fit, including *R*^2^ (0.9906), adjusted *R*^2^, predicted *R*^2^, and adequate precision. The high *R*^2^ value shows that 99% of the experimental data align with the model predictions, whereas a coefficient of variation of 2.86% confirms the model's reliability.

**Table 6:** Fit statistics

Std. dev.	Mean	C.V. %	*R* ^2^	Adjusted *R*^2^	Predicted *R*^2^	Adequate precision
2.03	70.85	2.86	0.9906	0.9819	0.9528	39.7701

### 3.3. Interactions between the process variables

**Fig. 4:**
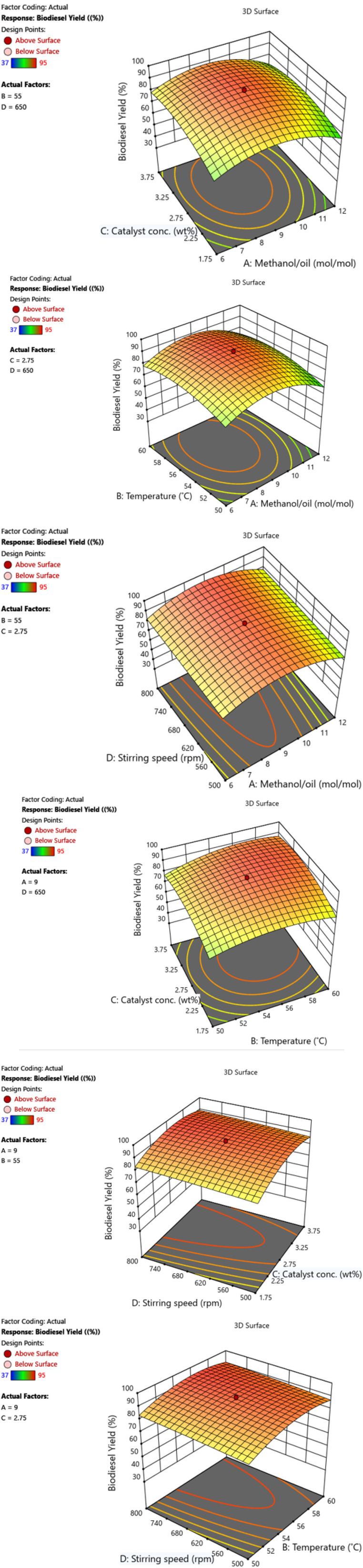
(a) Impact of catalyst concentration and methanol-to-oil molar ratio on biodiesel yield. (b) Impact of temperature and methanol-to-oil molar ratio on biodiesel yield. (c) Impact of the stirring speed and methanol/oil molar ratio on the biodiesel yield. (d) Impact of catalyst concentration and temperature on biodiesel yield. (e) Impact of stirring speed and catalyst concentration on biodiesel yield. (f) Effects of the stirring speed and temperature on the biodiesel yield.

Fig. 4 shows a 3D surface illustrating the combined effect of process variables on biodiesel yield. In Fig. 4(a), the influence of the methanol-to-oil molar ratio (6–12 mol mol^−1^) and catalyst concentration (1.75–3.75 wt%) was examined at a constant temperature of 55 °C and a stirring speed of 650 rpm. The biodiesel yield increased from 67% to 94% as the molar ratio increased from 6 to 9 and the catalyst concentration increased from 1.75 to 3.75 wt%. However, further increasing the molar ratio to 12 caused the yield to decrease to 65%.

A higher biodiesel yield was achieved with a 3.01 wt% catalyst concentration and an 8.75 : 1 methanol-to-oil molar ratio.^31^ Exceeding this optimal ratio led to emulsion formation, complicating biodiesel–glycerol separation and increasing costs. Additionally, excess methanol diluted the reaction mixture and reduced the catalyst efficiency.^32,33^

A higher biodiesel yield was achieved with a 3.01 wt% catalyst concentration and an 8.75 : 1 methanol-to-oil molar ratio. Low catalyst concentrations resulted in incomplete reactions or minimal biodiesel yields. However, increasing the catalyst concentration to the optimal level enhances biodiesel production.^34^ Conversely, when the catalyst concentration exceeded the optimum value, the soap and glycerol contents increased, causing a decrease in biodiesel production. The reactant viscosity increased with increasing catalyst concentration during the chemical reaction, which eventually decreased the biodiesel yield.^35^ In this investigation, the ideal methanol-to-oil mole ratio and concentration of the catalyst were found to be 8.75 : 1 and 3.01 wt%, respectively. This led to a 95% biodiesel yield.

Fig. 4(b) displays the 3D surface response, showing the combined effects of the methanol/oil molar ratio (6–12 mol mol^−1^) and temperature (50–60 °C) on the biodiesel yield, with the catalyst concentration fixed at 2.75 wt% and the stirring speed at 650 rpm. The yield increased from 60% to 92% as the molar ratio increased from 6 to 9 and the temperature increased from 50 °C to 60 °C. The higher temperature also enhanced the catalyst porosity and crystal growth, further increasing the yield. Another study reported that a 95% biodiesel yield could be achieved if the reaction was run at 55 °C and an 8 : 1 methanol-to-oil molar ratio.^36^

Fig. 4(c) shows the 3D surface response of the biodiesel yield influenced by the methanol–oil ratio (6–12 mol mol^−1^) and stirring speed (500–800 rpm), with a constant catalyst concentration (2.75 wt%) and temperature (55 °C). As the stirring speed increased, particularly at a 9 : 1 molar ratio, the yield rose from 87% to 92.5%. This improvement is attributed to enhanced mixing, which increases the reaction area and promotes more frequent collisions between reactants and catalyst surfaces, increasing biodiesel production.

Fig. 4(d) shows the 3D surface response to the combined effect of catalyst concentration (1.75–3.75 wt%) and temperature (50–60 °C) on biodiesel yield, with a fixed methanol/oil ratio of 9 mol mol^−1^ and a stirring speed of 650 rpm. The yield significantly increased from 71% to 92.5% as both the catalyst concentration and temperature increased, particularly between 1.75 and 3.75 wt% and between 50 °C and 55 °C.

Fig. 4(e) shows the 3D surface response of the biodiesel yield to the catalyst concentration (1.75–3.75 wt%) and stirring speed (500–800 rpm) at a constant temperature (55 °C) and methanol/oil ratio (9 mol mol^−1^). As the stirring speed increased from 500 to 800 rpm at a fixed catalyst concentration of 2.75 wt%, the yield increased notably from 80% to 91.5%.

Fig. 4(f) displays the 3D surface response of the biodiesel yield influenced by temperature (50–60 °C) and stirring speed (500–800 rpm), with a constant methanol–oil ratio (9 mol mol^−1^) and catalyst concentration (2.75 wt%). The yield increased from 83% to 90.3% as the stirring speed increased from 500 to 800 rpm at a fixed temperature of 55 °C.

### 3.4. Composition and physicochemical properties

Castor oil contains 0.72% palmitic acid, 0.95% stearic acid, 5.12% oleic acid, 4.43% linoleic acid, and 88% ricinoleic acid. Neem oil contains 3.70% palmitoleic acid, 23.72% palmitic acid, 11.86% stearic acid, 47.62% oleic acid, 8.74% linoleic acid and 3.44% linoleic acid. The FAME composition of biodiesel varies on the basis of its source. Saturated acids such as palmitoleic, palmitic, and stearic acids constitute 1.35%, 9.26%, and 6.32% of the total composition, respectively. However, unsaturated acids, including oleic acid, linoleic acid, and ricinoleic acid, make up 22.36%, 5.12%, and 43.91% of the composition, respectively. Following the two-step production process, the resulting methyl ester was subjected to detailed analysis to assess its physicochemical properties. The physicochemical parameters, such as acid value, pour point, density, cloud point, flash point, kinematic viscosity, and calorific value, met the standards outlined in ASTM D6751. The relative uncertainties of the measured parameters are listed in Table 7. The physicochemical attributes of the methyl esters are listed in Table 8. The density and kinematic viscosity of biodiesel have improved, as shown in Table 8. Thus, under ideal circumstances, the generated biodiesel can serve as a viable alternative to traditional Petro-diesel.

**Table 7:** Relative uncertainty of the measured parameters considering instrument accuracy for the average diesel value

Parameter	Relative uncertainty	Instrument accuracy	The average value for diesel
Density	±0.1/812 = 0.000123	±0.1 kg m^−3^	812
Kinematic viscosity	±0.1/2.40 = 0.0416	±0.1 mm^2^ s^−1^	2.40
Acid value	±0.001/0.07 = 0.0142	±0.001 mg KOH per g	0.07
Calorific value	±0.1/45.5 = 0.00219	±0.1 MJ kg^−1^	45.5
Flash point	±0.1/77 = 0.00129	±0.1 °C	77
Pour point (°C)	±0.1/12 = 0.00833	±0.1 °C	−12
Cloud point (°C)	±0.1/9 = 0.01111	±0.1 °C	−9
Fire point (°C)	±0.1/83 = 0.00121	±0.1 °C	83
BP	±0.025/3.5 = 0.00714	±0.025 kW	3.5
BSFC	±0.04/276 = 0.000144	±0.04 g kW^−1^ h^−1^	276
BTE	±0.4/28.9 = 0.01384	±0.4	28.9
EGT	±1/222.5 = 0.00449	±1 °C	222.5
CO	±0.015/0.13 = 0.11538	±0.015 vol%	0.13
CO_2_	±0.015/5.07 = 0.00295	±0.015 vol%	5.07
NOx	±1/128.21 = 0.00779	±1 ppm	128.21
Smoke opacity	±0.015/7.78 = 0.00192	±0.015 vol%	7.78

**Table 8:** Properties of the fuels and comparison with the ASTM standard values

Sr./no.	Properties	B10	B20	B30	Biodiesel 100	Diesel fuel	ASTM D6751
1	Density (kg m^−3^)	820	830	841	910	812	880
2	Kinematic viscosity at 40 °C (mm^2^ s^−1^)	2.92	3.48	3.95	7.82	2.40	1.9–6
3	Calorific value (MJ kg^−1^)	44.6	43.9	43.2	38.8	45.5	—
4	Acidic number (mg KOH per g)	0.092	0.107	0.120	0.21	0.07	Max 0.5
5	Flash point (°C)	82.4	90.7	99.4	199	77	Min 130
6	Pour point (°C)	−11.45	−11.15	−10.90	−4	−11.9	—
7	Fire point (°C)	86	92	99	202	83	—
8	Cloud point (°C)	−8.80	−8.71	−8.58	−8	−9	—
9	Cetane number	54.52	54.88	54.26	54.34	50.18	47 min

### 3.5. Performance

#### 3.5.1. Brake specific fuel consumption

**Fig. 5:**
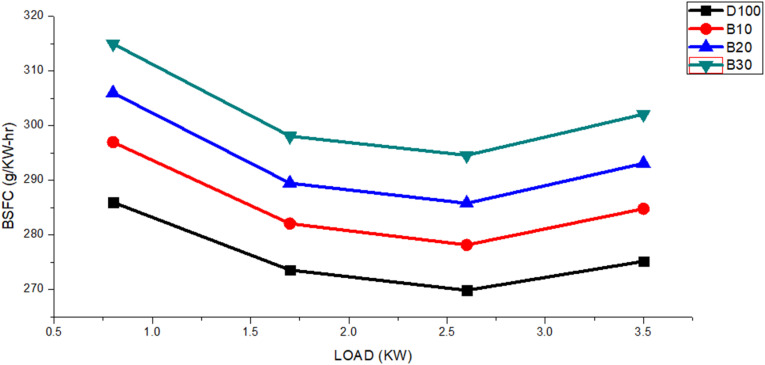
BSFC variants for the DF100, B10, B20, and B30 fuels with an engine load.

BSFC, or brake-specific fuel consumption, is a crucial metric for assessing the efficiency with which an engine makes use of the fuel that is provided to produce energy. The BSFC values of biodiesel blended fuels are shown in Fig. 5, along with a comparison with those of pure diesel fuel. Changes in these values were observed with engine load. The figure shows that as the engine load increased from 0.8 to 3.5 kW. However, notably, the BSFC for these blends remained greater than that of pure diesel. Specifically, the maximum BSFC values for DF100, B10, B20, and B30 were 286, 297, 306, and 315 g kW^−1^ h^−1^, respectively. The average BSFC values for fuels DF100, B10, B20, and B30 were 276, 293, 285, and 302 g kW^−1^ h^−1^, respectively. The average BSFC for DF100 was consistently 3.37%, 6.29%, and 9.51% lower than those of B10, B20, and B30, respectively.

Its high density, high viscosity, and low calorific value are the main variables affecting biodiesel use.^37,38^ However, the low calorific value is the key factor that contributes to increased biodiesel blended fuel.^39^ During combustion, the calorific value indicates the energy potential within the fuel, with a higher value corresponding to greater energy yield.^40^ The figure shows that all biodiesel blends have a lower calorific value, primarily due to the presence of oxygen molecules in the fuel. This reduction delays combustion onset, increases fuel consumption,^41^ and decreases energy release, leading to reduced piston pressure and, consequently, less usable work.^42^ Additionally, poor atomization and uneven combustion are caused by the high density and viscosity of fuel.^43^

#### 3.5.2. Brake thermal efficiency

**Fig. 6:**
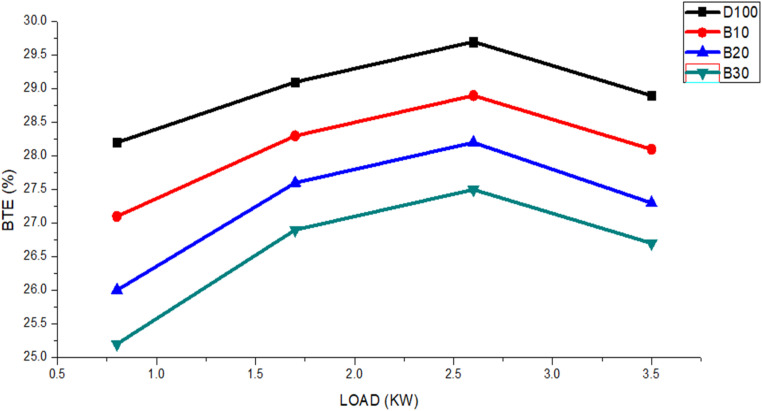
BTE variants for the DF100, B10, B20, and B30 fuels with an engine load.

The fuel economy of an internal combustion engine is evaluated in terms of brake thermal efficiency. The relationship between BTE and the BSFC is inverse.^44^ The BTE values of the biodiesel blended fuels are shown in Fig. 6, along with a comparison with those of pure diesel fuel. With respect to engine load, variations in these values were noted. The figure shows that as the engine load increased from 0.8 to 3.5 kW. However, notably, the BTE of these blends remained lower than that of pure diesel. Specifically, the BTE values for fuels DF100, B10, B20, and B30 were 28.2%, 27.7%, 26%, and 25.2%, respectively. The average BTE values for fuels DF100, B10, B20 and B30 were 28.9%, 28.1%, 27.3%, and 26.5%, respectively. Additionally, the average brake thermal efficiency of DF100 was consistently 3.02%, 5.88%, and 8.31% greater than those of B10, B20, and B30, respectively.

The density and viscosity of biodiesel were found to be greater than those of pure diesel fuel, and this discrepancy significantly impacted engine performance. The elevated density and viscosity of biodiesel blended fuel had notable effects on the engine's fuel injection system, including alterations in injection timing, changes in the injected spray pattern, and adjustments in the injection fuel amount.^45^ The high density and viscosity of blended biodiesel fuel hinder atomization and vaporization,^46^ preventing effective droplet formation and prolonging mixing with air.^47^ They also impede smooth fuel flow in the combustion chamber, leading to delayed combustion and ultimately reduced engine power output.^48^

#### 3.5.3. Exhaust gas temperature

**Fig. 7:**
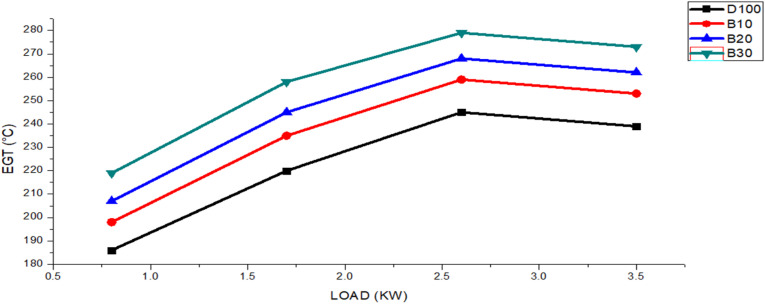
EGT variants for the DF100, B10, B20, and B30 fuels with an engine load.

The exhaust gas temperature helps determine how effectively combustion occurs inside the chamber.^49^ EGT serves as a critical parameter, as it allows us to deduce the in-cylinder temperature, and at elevated temperatures, exhaust gases undergo reactions to produce new substances.^44,50^ The EGT values of the biodiesel blended fuels are shown in Fig. 7, along with a comparison with those of pure diesel fuel. With respect to engine load, variations in these values were noted. The figure shows that as the engine load increased from 0.8 to 3.5 kW. However, notably, the EGT for these blends remained higher than that of neat diesel. Specifically, the maximum EGT values for DF100, B10, B20, and B30 were 298, 310, 323 and 333 °C, respectively. The average EGT values for fuels DF100, B10, B20, and B30 were 222.5, 236.3, 245.5, and 257.2 °C, respectively. Additionally, the average EGT for DF100 was consistently 6.21%, 10.41%, and 15.77% lower than those of B10, B20, and B30, respectively.

As the engine load increases, the EGT value increases because of the need to increase the fuel capacity to overcome additional loads while maintaining the same speed.^51^ When we compared the EGT values of pure diesel and various biodiesel blended fuels, we observed that the EGT for the biodiesel blended fuels exceeded that of pure diesel, primarily because of the increased fuel consumption during the combustion process.^52^ The cetane number, a key indicator of fuel ignition quality, contributed significantly to the influence of the EGT value. Biodiesel, with its higher cetane number, ignited closer to the injector, leading to increased heat generation, reduced ignition delay, and ultimately greater EGT.^53,54^

### 3.6. Emissions

#### 3.6.1. CO emissions

**Fig. 8:**
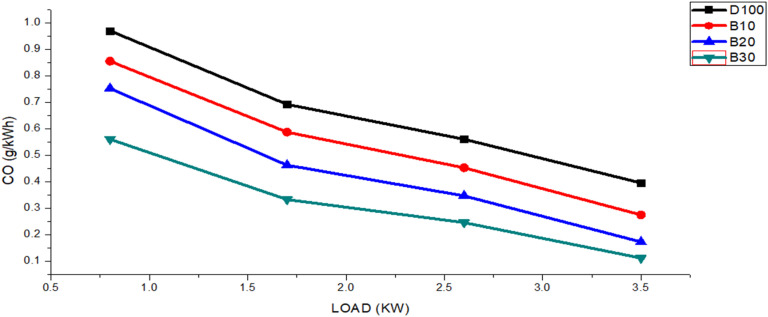
The CO emissions of the DF100, B10, B20, and B30 fuels vary with engine load.

The indication of incomplete fuel combustion is the presence of CO emissions in exhaust within a combustion chamber, which is attributable to insufficient oxygen content.^55^Fig. 8 shows the CO emission results for various engine loads. The figure shows that as the engine load rose from 0.8 to 3.5 kW, all biodiesel blended fuels produced lower CO emissions than did neat diesel. The average CO emission values for fuels DF100, B10, B20, and B30 were 0.65, 0.54, 0.43, and 0.31 g kW^−1^ h^−1^, respectively. Additionally, the average CO emissions for DF100 were consistently 18.38%, 38.22%, and 56.31% greater than those of B10, B20, and B30, respectively.

When petroleum diesel was used, relatively high carbon monoxide emissions were observed due to its lack of oxygen, leading to incomplete combustion. Carbon monoxide formation is also influenced by factors such as the air–fuel ratio, fuel type, combustion chamber design, atomization rate, injection pressure, injection timing, engine load, and speed.^43^ In contrast, biodiesel produced lower carbon monoxide emissions because its inherent oxygen content promotes more complete combustion.^56,57^

#### 3.6.2. CO_2_ emissions

**Fig. 9:**
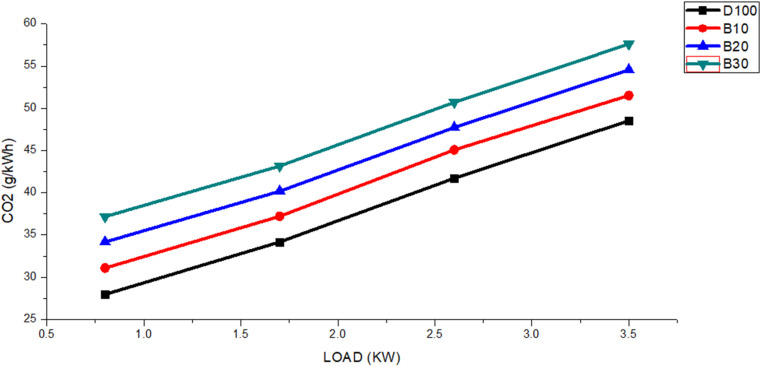
The DF100, B10, B20, and B30 fuels exhibit variations in CO_2_ emissions with engine load.

CO_2_ formation occurs within a combustion chamber when an ample quantity of oxygen is available to facilitate the complete combustion of fuel. Fig. 9 shows the CO_2_ emission results for various engine loads. The figure shows that as the engine load rose from 0.8 to 3.5 kW, all blended biodiesel fuels produced greater CO_2_ emissions than did pure diesel. The average CO_2_ emission values for fuels DF100, B10, B20, and B30 were 38.09, 41.22, 44.15, and 47.16 g kW^−1^ h^−1^, respectively. Additionally, the average CO_2_ concentration of DF100 was consistently 8.56%, 16.61%, and 24.88% lower than those of B10, B20, and B30, respectively.

The emission of CO_2_ results from the thorough combustion of fuel, facilitated by the elevated temperatures within the engine cylinder.^58^ An additional reason for the increased CO_2_ emissions is the higher oxygen content in biodiesel, which enhances combustion and promotes greater conversion of carbon monoxide to carbon dioxide.

#### 3.6.3. NOx emissions

NOx formation commonly arises through three distinct mechanisms: thermal NOx generation, prompt NOx generation, and fuel NOx mechanisms. In the intricate processes of the fuel NOx mechanism, nitrogen in the fuel undergoes oxidation, resulting in the generation of NOx. Nevertheless, given the remarkably scant natural nitrogen content found in both diesel and biodiesel, the contribution of the fuel NOx formation mechanism is generally deemed inconsequential.^59^ Within the thermal NOx mechanism, the genesis of NOx emissions arises from a sequence of chemical reactions between N_2_ and O_2_ stimulated by elevated combustion temperatures, referred to as the Zeldovich mechanism. This mechanism predominates in contributing to overall NOx formation during diesel engine combustion on the basis of the principles elucidated by the Zeldovich mechanism. The sequence of reactions that make up the Zeldovich mechanism is described through eqn (7)–(9).^60^

7 O + N_2_ ⇌ NO + N

8 N + O_2_ ⇌ NO + O

9 N + OH ⇌ NO + H

These chemical reactions occurring within diesel engines lead to the production of NO. The NO generated within the flame zone undergoes conversion into NO_2,_ and a specific (10) equation is outlined.

10 NO + OH_2_ ⇌ NO_2_ + 0H

Following this, the transformation of NO_2_ back to NO takes place according to a particular (11) equation.

11 NO_2_ + O ⇌ NO + O_2_

The Zeldovich mechanism is highly sensitive to temperature in terms of the NO production rate, with elevated temperatures corresponding to increased NOx emissions.^61^ In a prompt NOx mechanism, the combustion process involves the reaction of hydrocarbon fragments (CH and CH_2_) with N_2_. This reaction yields species containing C–N, which subsequently undergo further reactions with O_2_, leading to the production of NOx. Prompt NOx is also known as Fenimore NOx. Eqn (12) prompts the mechanism's reaction to be expressed as.^50^

12 CH + N_2_ ⇌ HCN + N

The increased production of hydrocarbons occurs during the combustion of unsaturated FAME in contrast to regular diesel, resulting in elevated NOx formation. Moreover, prompt NOx becomes particularly noticeable under fuel-rich circumstances, characterized by an abundance of hydrocarbon fragments available for interaction with N_2_.^62^ Prompt and thermal mechanisms significantly contribute to generating NOx during the combustion of biodiesel.^63^

**Fig. 10:**
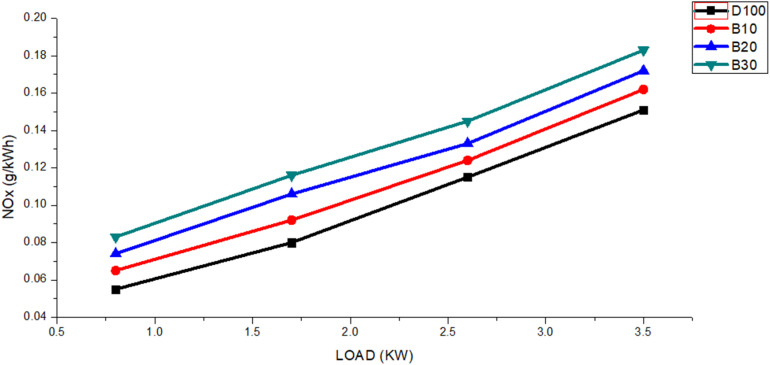
The DF100, B10, B20, and B30 fuels exhibit variations in NOx emissions with engine load.

Fig. 10 shows the NOx emission results for various engine loads. The figure shows that as the engine load rose from 0.8 to 3.5 kW, all blended biodiesel fuels produced greater NOx emissions than did pure diesel. The average NOx emission values for fuels DF100, B10, B20, and B30 were 0.11, 0.112, 0.122 and 0.131 g kW^−1^ h^−1^, respectively. Additionally, NOx emissions for DF100 were consistently 14.01%, 24.57%, and 36.25% lower than those for B10, B20, and B30, respectively. When biodiesel was utilized, the engine combustion process experienced a shorter igniting delay due to the increased oxygen content compared with that of neat diesel. As a result, NOx emissions are higher in biodiesel blended fuel.^64^

#### 3.6.4. Smoke opacity

**Fig. 11:**
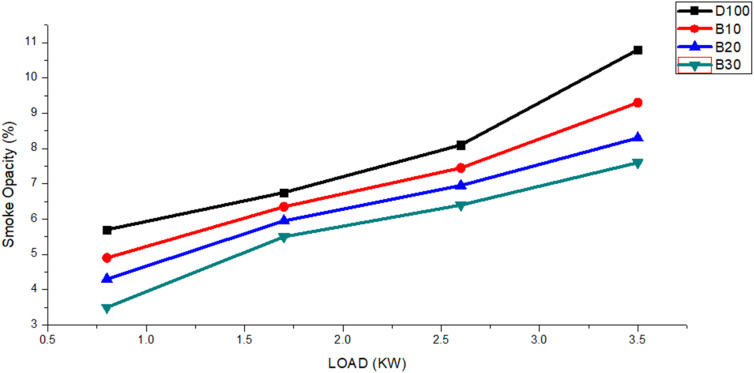
The smoke opacity of the DF100, B10, B20, and B30 fuels varies with engine load.

The measurement of smoke opacity serves as a gauge for the quantity of smoke emitted by a diesel engine. The primary factor influencing smoke opacity is the bound oxygen content within the fuel.^65^ Smoke opacity is predominantly related to the amount of soot generated by fuels while they are undergoing combustion.^66^Fig. 11 shows the smoke opacity results for various engine loads. The figure shows that as the engine load rose from 0.8 to 3.5 kW, all blended biodiesel fuels produced lower smoke opacity than did neat diesel. The average smoke opacity values for fuels DF100, B10, B20 and B30 were 7.8%, 7.1%, 6.3%, and 5.7%, respectively. Additionally, the average smoke opacity of DF100 was consistently 10.46%, 18.43% and 26.93% greater than those of B10, B20, and B30, respectively.

The combustion of petroleum diesel fuel results in more carbon-based substances, primarily due to the incomplete burning of hydrocarbons (HCs).^67^ Higher biodiesel blends reduce smoke opacity by lowering carbon content and enhancing oxygen availability during combustion.^68^

## 4. Conclusion

A central composite design is utilized to maximize the characteristics of the production process. This investigation details the usage of nonedible oils; specifically, a blend of neem and castor oils provides a feasible way to produce high-quality biodiesel that can be used as fuel. The production process involves a method comprising transesterification. Subsequently, 95% biodiesel production was obtained at 56.6 °C, an 800 rpm agitation speed, and a methanol/oil molar ratio of 8.75 : 1, with 3.01 wt% calcium oxide. The variables examined during transesterification reactions demonstrated a notable effect on the yield percentage of biodiesel. Finally, the produced biodiesel was examined *via* the standard values provided by EN 14214 and ASTM D6751, and the density, kinematic viscosity, pour point, and cloud point of biodiesel were improved, which demonstrated that it fulfilled all the requirements and increased its potential as a substitute fuel source for diesel engines.

Engine tests revealed that the BSFC initially decreased and then increased as the load on the engine increased. The engine test revealed that BTE initially increased but then decreased as the load on the engine increased. As the load increased and the blend ratio of biodiesel increased, engine testing revealed that the EGT increased. For all biodiesel blended fuels, experimental work indicated that CO emissions were lower than those of neat diesel. The average CO emissions for DF100 were consistently 18.38%, 38.22%, and 56.31% greater than those of B10, B20, and B30, respectively. Additionally, the average smoke opacity of DF100 was consistently 10.46%, 18.43%, and 26.93% greater than those of B10, B20, and B30, respectively. With every combination of biodiesel fuel, experimental work indicated that the emissions of CO_2_ exceeded those of pure diesel. Experimental work has shown that NOx emissions are greater than those of pure diesel for all biodiesel blended fuels. On the basis of an overall assessment, a biodiesel blend from castor and neem oils can be a viable substitute fuel for internal combustion engines.

## Conflicts of interest

The authors declare that they have no known competing financial interests or personal relationships that could have appeared to influence the work reported in this paper.

## Data Availability

The data supporting this article have been included as part of the SI.
